# A bittersweet symphony: soluble mediator networks orchestrate melanoma plasticity and therapeutic adaptation

**DOI:** 10.3389/fcell.2026.1905416

**Published:** 2026-09-25

**Authors:** Carmine Lauretta, Carmela Sorrentino, Emilia Sabbatino, Viviana Tutino, Deborah Simão de Souza, Antimo Migliaccio, Gabriella Castoria, Pia Giovannelli, Marzia Di Donato

**Affiliations:** 1 Department of Precision Medicine, University of Campania “L. Vanvitelli”, Naples, Italy; 2 Department of Pharmacology, Laboratory of Experimental Endocrinology, Escola Paulista de Medicina (EPM), Universidade Federal de São Paulo (UNIFESP), São Paulo, Brazil

**Keywords:** melanoma, phenotypic plasticity, soluble mediators, therapeutic resistance, tumor microenvironment

## Abstract

Melanoma progression and therapeutic resistance are increasingly recognized as emergent properties of the dynamic interactions between tumor cells and their surrounding tumor microenvironment (TME). A central feature of this adaptability is melanoma cell plasticity, through which tumor cells reversibly transition between distinct transcriptional and functional states in response to microenvironmental and therapeutic pressures. Recent advances in single-cell RNA sequencing and spatial transcriptomics have revealed that these interactions are spatially organized, with distinct cellular compositions and signaling programs forming specialized microenvironmental niches. However, the mechanisms that coordinate these spatially and functionally heterogeneous states remain incompletely integrated. In this review, we propose that soluble mediator networks, including cytokines, chemokines, growth factors, metabolites, extracellular vesicle-associated signals, and stress-related mediators, provide a functional layer that links the different compartments of the melanoma TME. We organize this network into interconnected immunoregulatory, vascular and lymphatic, mechanotransductive/ECM, and metabolic/redox axes, and examine how their reciprocal interactions influence melanoma cell identity, immune surveillance, vascular function, tissue architecture, and metabolic adaptation. Particular attention is given to the context-dependent activity of soluble mediators, whose effects vary according to their cellular source, spatial distribution, disease stage, and therapeutic pressure. We further discuss how these signals converge within localized microenvironmental niches to promote melanoma plasticity, immune escape, metastatic dissemination, and therapeutic resistance. Emerging mechanisms, including metabolic rewiring, lactate signaling, oxidative stress, and transmissible endoplasmic reticulum stress, illustrate how adaptive information can propagate between the different compartments. Overall, we propose that therapeutic resistance should not be viewed solely as the consequence of tumor-intrinsic alterations or isolated resistance pathways, but as an emergent property of a continuously rewired tumor ecosystem. This perspective suggests that effective therapeutic strategies may require disruption of the adaptive communication networks that sustain resistant tumor states, rather than targeting individual molecular pathways in isolation.

## 1 Introduction

Cutaneous melanoma remains the most lethal form of skin cancer, associated with a high mortality rate (Sung et al., 2026). Its progression is fundamentally governed not only by tumor-intrinsic genetic alterations but also by continuous, dynamic interactions between malignant cells and the surrounding tumor microenvironment (TME) (Kuras, 2023). The melanoma TME is composed of heterogeneous stromal, immune, vascular, and neural elements embedded within a structurally and biochemically active extracellular matrix (ECM) (Hossain and Eccles, 2023; Kharouf et al., 2023).

The therapeutic landscape of advanced melanoma has been profoundly transformed by the introduction of targeted therapies, specifically combined BRAF/MEK inhibition, and immune checkpoint inhibitors (ICIs) targeting the PD-1/PD-L1, CTLA-4, and LAG3 pathways. While these regimens have significantly improved patient survival, primary resistance and acquired therapeutic failure remain critical clinical hurdles (Marine et al., 2020; Fujisawa et al., 2021; Patel et al., 2021). This therapeutic evasion is increasingly recognized as an emergent property of microenvironmental adaptation. At the core of this process is the phenotypic plasticity of melanoma cells, which continuously and reversibly shift between distinct transcriptional and functional states to survive microenvironmental pressures such as hypoxia, nutrient deprivation, immune surveillance, and drug-induced stress (Rambow et al., 2019; Hu et al., 2025).

Recent advances in single-cell RNA sequencing (scRNA-seq) and spatial transcriptomics have demonstrated that melanoma is organized into specialized multicellular niches characterized by distinct cellular compositions, transcriptional programs, and soluble mediator landscapes (Pelka et al., 2021). Single-cell resolution transcriptomic profiling has identified highly heterogenous immune and stromal populations within these niches, including TCF7^+^ stem-like CD8^+^ T cells associated with durable immunotherapy responses and terminally exhausted TOX^+^ T-cell populations linked to immune dysfunction (Sade-Feldman et al., 2018). Similarly, cancer-associated fibroblasts (CAFs) include inflammatory CAFs (iCAFs) secreting immunomodulatory cytokines (e.g., IL-6, CXCL12) and myofibroblastic CAFs (myCAFs) that predominantly drive ECM deposition and mechanotransduction (Biffi and Tuveson, 2021). Spatial analyses further revealed that immune-infiltrated regions coexist with immune-excluded niches characterized by dense ECM deposition, metabolic reprogramming, suppressive myeloid populations and stromal barriers (Jerby-Arnon et al., 2018). In this context, therapy-resistant melanoma states often colocalize with exhausted lymphocytes and immunosuppressive macrophages, generating localized microanatomical niches that support resistance to therapy (Nirmal et al., 2022). While these recent multi omics technologies have successfully mapped the cellular atlas of these resistant niches (Tirosh et al., 2016), the underlying communication mechanisms remain to be fully explained.

This review aims to establish a conceptual framework where soluble mediators, encompassing cytokines, chemokines, growth factors, metabolites, lipid mediators, extracellular vesicle (EV)-associated soluble cargo, and stress-associated signaling molecules, constitute the primary functional layer integrating different compartments into interconnected, self-regulating circuits (Lázár-Molnár et al., 2000; Falcone et al., 2020; Xu et al., 2024).

This soluble network has a functional duality, or “bittersweet” nature: the mediators rarely exert unidirectional biological effects. Instead, spatial organization, temporal dynamics, and therapeutic context dictate whether a specific signaling circuit restrains or promotes melanoma progression, as resumed in Table 1.

**TABLE 1:** Context-dependent functional duality of representative soluble mediator networks: the biological basis of the “Bittersweet Symphony”.

Biological axis	Representative soluble mediator(s)/signaling network	Physiological/homeostatic role	Tumor-promoting role in melanoma	Determinants of the functional switch	References
ECM remodeling/Stromal remodeling	TGF-β	Tissue repair and ECM homeostasis	CAF activation, ECM stiffening, dedifferentiation and immune suppression	Chronic TGF-β signaling, fibrosis, hypoxia	(Falcone et al., 2020; Mao et al., 2021; Hossain and Eccles, 2023; Zhou et al., 2024)
Immune regulation	IFN-γ	Activates CD8^+^ T cells and NK cells, promotes antigen presentation and immune surveillance	Chronic IFN-γ signaling induces adaptive immune resistance, PD-L1 expression and T-cell exhaustion	Persistent inflammatory signaling, therapeutic pressure	(Balsamo et al., 2009; Spranger et al., 2015; Jedlička et al., 2022; Zheng et al., 2024)
Inflammatory signaling	TNF-α	Coordinates acute inflammatory responses, promotes pathogen clearance, and supports anti-tumor immunity through activation of innate and adaptive immune cells	Sustains chronic inflammation, melanoma cell plasticity, immune suppression, and therapeutic resistance by promoting a persistent pro-tumor inflammatory microenvironment	Chronic TNF-α signaling, together with persistent inflammation, reciprocal tumor–stroma interactions, hypoxia, and sustained inflammatory feedback loops within the melanoma TME, shifts TNF-α activity from acute immune defense to chronic tumor-promoting inflammation	(Elias et al., 2010; Smith et al., 2014; Xu et al., 2024)
Angiogenesis	VEGF	Physiological angiogenesis during tissue repair	Dysfunctional angiogenesis, immune exclusion and therapy resistance	Chronic hypoxia, sustained VEGF signaling	(Viallard and Larrivée, 2017; Wu et al., 2022; Cazzato et al., 2024; Leone et al., 2024)
Redox signaling/Oxidative stress	ROS	Acute or moderate oxidative stress promotes the expression of stress-induced ligands and danger signals, enhancing immune recognition and NK-cell-mediated cytotoxicity	Oxidative adaptation, TERS, immune suppression and therapy resistance	Persistent oxidative stress driven by hypoxia, oxygen deprivation, nutrient imbalance, metabolic stress, and chronic UPR activation reinforces adaptive stress signaling and melanoma survival within the TME	(Semenza, 2012; Almeida et al., 2019; Pizzimenti et al., 2021; Ni et al., 2025; Sorrentino et al., 2026)
Innate immune sensing	STING signaling	Type I IFN production, DC maturation and T-cell priming	Chronic STING activation sustains inflammation, immune dysfunction and tumor adaptation	Persistent or spatially dysregulated STING activation together with suppressive myeloid cells and metabolic constraints within the melanoma TME	(Woo et al., 2014; Corrales et al., 2015; Sivick et al., 2018; Li et al., 2019)
Metabolic rewiring	Lactate	Physiological metabolic intermediate supporting cellular metabolism	Immunosuppression, angiogenesis and metabolic adaptation	Aerobic glycolysis, hypoxia, poor perfusion	(Fischer et al., 2007; Avagliano et al., 2020; Li et al., 2022; Apostolova and Pearce, 2022; Zheng et al., 2024)
ER stress/UPR	ER stress/UPR signaling	Restores proteostasis and cellular homeostasis under transient stress conditions	Sustains metabolic adaptation, transmissible ER stress (TERS), immune dysfunction, and therapeutic resistance	Persistent hypoxia, oxidative stress, nutrient deprivation, and chronic activation of the UPR shift ER stress signaling from adaptive recovery toward ecosystem-wide tumor adaptation	(Mahadevan et al., 2011; Lebeaupin et al., 2020; Ong and Logue, 2023; Hwang et al., 2025)

Consequently, therapeutic resistance cannot be decoupled from microenvironmental rewiring (Visser and Joyce, 2023). While several comprehensive reviews have described the cellular composition and molecular complexity of the melanoma TME or focused on individual processes such as immunity, angiogenesis, ECM remodeling, and metabolic adaptation, we propose a complementary conceptual model. By placing soluble mediator networks at the centre, we do not consider them as parallel entities, but we track how these localized signaling circuits cross-evaluate mechanical cues, metabolic stress, and immune exclusion to sustain tumor cell survival. Ultimately we aim to provide a biological rationale for combinatorial strategies designed to simultaneously disrupt multiple adaptive communication pathways.

## 2 The melanoma microenvironment as an integrated network

Melanoma TME comprises an interconnected network of stromal, vascular, immune, and ECM components. Major stromal cell populations, including CAFs, tumor-associated endothelial cells (TECs), pericytes (PCs) and mesenchymal stromal cells contribute to ECM remodeling, angiogenesis, and metabolic rewiring. In addition, immune cells such as tumor-associated macrophages (TAMs), regulatory T cells (Tregs), dendritic cells (DCs), CD8^+^ T cells, and natural killer (NK) cells (Simiczyjew et al., 2020) dynamically evolve during melanoma progression and in response to therapeutic pressure. However, the melanoma TME is also full of an extensive network of soluble mediators, including pro-inflammatory cytokines (IL6, TNF-α, IL-1α, IL-1β), immunosuppressive cytokines (IL-10, TGF-β), growth factors (VEGF, FGF, EGF), and chemokines (CCL2, CCL5, CXCL12) (Elias et al., 2010; Xu et al., 2024).

In addition to their transcriptional effects, soluble mediators progressively reshape tissue organization, immune function, and cellular behaviour, thereby sustaining chronic inflammation, matrix remodeling, immune escape, and therapeutic resistance. Their biological activity is highly context-dependent and may exert either tumor-promoting or tumor-restraining functions depending on spatial organization, disease stage, and therapeutic context (Negovetić et al., 2026). The melanoma TME should therefore be viewed less as a static collection of specialized cell types and more as a self-organizing biological system in which soluble mediators continuously redefine cellular identity, functional relationships, and tissue architecture. Accordingly, therapeutic resistance should not be interpreted as the consequence of alterations within a single cellular compartment but rather as an emergent property arising from the collective behavior of the entire signaling network. Figure 1 summarizes how soluble mediator networks orchestrate melanoma plasticity across the TME. The principal cellular and non-cellular components, together with key soluble mediators and associated biological functions, are summarized in Table 2.

**FIGURE 1:**
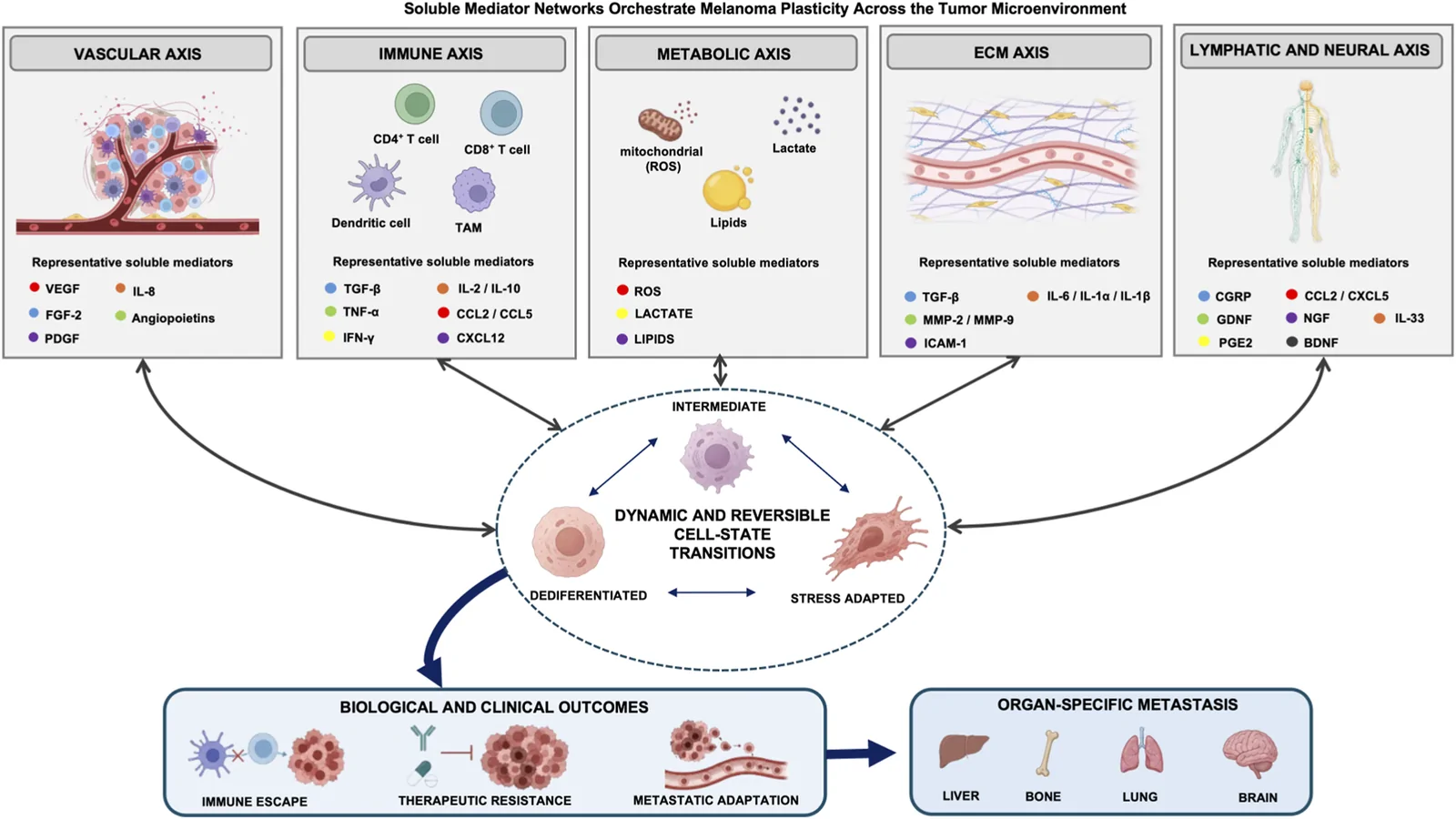
Soluble mediator networks orchestrate melanoma plasticity across the tumor microenvironment. The schematic illustrates the dynamic and bidirectional communication network operating within the TME, highlighting how soluble mediators integrate distinct biological compartments into an adaptive ecosystem rather than acting through independent signaling pathways. (Top) The principal biological axes are shown together with representative cellular components and key soluble mediators coordinating their reciprocal communication. (Center) Signals originating from the different TME compartments converge to promote melanoma plasticity through dynamic and reversible cell-state transitions, allowing continuous phenotypic remodeling and functional adaptation in response to microenvironmental and therapeutic cues. (Bottom) The coordinated activity of these interconnected networks drives the major biological and clinical outcomes of melanoma progression, including immune escape, therapeutic resistance, metastatic dissemination, ultimately promoting the establishment of organ-specific metastatic niches that support colonization and outgrowth in distant organs, including the brain, lung, liver, and bone. BDNF, brain-derived neurotrophic factor; CCL/CXCL, chemokine ligands; CD, cluster of differentiation; CGRP, calcitonin gene-related peptide; ECM, extracellular matrix; FGF-2, fibroblast growth factor 2; GDNF, glial cell line-derived neurotrophic factor; ICAM-1, intercellular adhesion molecule 1; IFN-γ, interferon gamma; IL, interleukin; MMP, matrix metalloproteinase; NGF, nerve growth factor; PDGF, platelet-derived growth factor; PGE2, prostaglandin E2; ROS, reactive oxygen species; TAM, tumor-associated macrophage; TGF-β, transforming growth factor beta; TNF-α, tumor necrosis factor alpha; VEGF, vascular endothelial growth factor.

**TABLE 2:** Soluble mediator networks coordinating melanoma plasticity across the major TME compartments.

TME compartment	Cellular/Non-cellular component	Dominant soluble mediators	Adaptive major biological functions	Contribution to melanoma plasticity	References
Tumor Compartment	Melanoma Cells	VEGF, FGF-2, PDGF, IL-8; TGF-β, EGF, CCL2; CCL5, CXCL12; extracellular vesicles (EVs), Angiopoietin-2	Angiogenesis induction and pre-metastatic niche establishment	Phenotypic switching and cell-state transitions	(Wu et al., 2022; Cazzato et al., 2024; Mayasin et al., 2024)
Stromal Compartment	Cancer-Associated Fibroblasts (CAFs), PCs, MSCs	IL-1α, IL-1β, IL-6; HGF; TGF-β; PGE2, MMPs (MMP-9); L-Arginine depletion	ECM deposition, NK/T cell suppression, and targeted/immune therapy resistance	Dedifferentiation and invasive reprogramming	(Balsamo et al., 2009; Érsek et al., 2020; Jurj et al., 2022)
Stromal Compartment	Mast Cells (MCs)	VEGF, FGF-2, IL-6, tryptase, chymase; MMPs	Neovascularization, ECM remodeling, and stromal stiffness enhancement	Pro-angiogenic remodeling and stromal priming	(Cerro et al., 2022)
Stromal Compartment	Senescent Fibroblasts	ROS-modulating proteins	ROS-mediated drug resistance via MAPK/PI3K modulation	SASP-driven adaptive reprogramming	(Almeida et al., 2019; Pizzimenti et al., 2021)
Immune Compartment (Suppressive)	Tumor-Associated Macrophages (TAMs)	IL-10, TGF-β, VEGF, matrix-remodeling enzymes; TNF-α	Cytotoxic T cell suppression, angiogenesis support, and MAPK inhibitor resistance	Immune evasion and therapy resistance	(Smith et al., 2014; Simiczyjew et al., 2020 Sikorski et al., 2025)
Immune Compartment (Suppressive)	Myeloid-Derived Suppressor Cells (MDSCs)	IL-10, TGF-β, NO, arginase-1	Inhibit T and NK cell activity via amino acid depletion and immunosuppressive cytokines	Immunosuppressive myeloid polarization	(Steinberg et al., 2017; Huber et al., 2018; Li et al., 2021)
Immune Compartment (Suppressive)	Regulatory T Cells (Tregs)	IL-10, TGF-β; IL-2 (metabolic competition)	Effector T cell inhibition and immune escape via metabolic competition	Immune tolerance maintenance	(Marshall et al., 2020; Simiczyjew et al., 2020; Liu et al., 2021)
Immune Compartment (Effector)	Cytotoxic CD8^+^ T Cells and Natural Killer (NK) Cells	Perforin, granzyme, IFN-γ	Melanoma cell elimination and pro-inflammatory signaling	Cancer immunoediting	(Simiczyjew et al., 2020; He et al., 2024)
Vascular Compartment	Tumor-Associated Endothelial Cells (TECs)	ANGPT2/TIE2 signaling; VE-cadherin, EphA2	Immune trafficking regulation, vasculogenic mimicry, and anti-angiogenic resistance	Vascular plasticity and immune exclusion	(Rigamonti et al., 2014; Leong and Kim, 2020; Serratì et al., 2025)
Biomechanical Niche	Extracellular Matrix (ECM)	MMP-2, MMP-9; Tissue Inhibitors of Metalloproteinases (TIMPs)	Structural support, physical barrier degradation, and metastatic modulation	Mechanotransduction-driven phenotypic reprogramming	(Kessenbrock et al., 2010; Pittayapruek et al., 2016)

## 3 The immunoregulatory axis: Orchestrating immune exclusion and suppression

Immune cell composition alone poorly predicts immune competence, as local cytokines, chemokines, and metabolic signals dynamically reprogram immune cell activation, differentiation, and exhaustion states, ultimately promoting metastatic dissemination and therapeutic resistance (Simiczyjew et al., 2020; Liu et al., 2021; Sikorski et al., 2025). Consequently, the same immune cell subset may exert tumor-restraining or tumor-promoting functions depending on the surrounding signaling context.

TAMs and myeloid-derived suppressor cells (MDSCs) represent major immunosuppressive populations within this network. TAMs are frequently polarized toward an M2-like phenotype through melanoma-derived cytokines and chemokines, establishing bidirectional feed-forward inflammatory loops that reinforce immune suppression and tumor progression (Simiczyjew et al., 2020; Sikorski et al., 2025). Concurrently, MDSCs suppress T- and NK-cell activity through arginase-1–mediated amino acid depletion, nitric oxide (NO) production, and release of immunosuppressive cytokines such as IL-10 and TGF-β. In addition, tumor-derived microRNAs further contribute to MDSCs expansion and adaptive resistance to immunotherapy (Steinberg et al., 2017; Huber et al., 2018).

In addition, Tregs further stabilize the immunosuppressive architecture of the melanoma TME. Their accumulation within melanoma lesions is driven by chemokine gradients, particularly via CCR4-dependent pathways, representing a clinically relevant mechanism for immune escape (Marshall et al., 2020). Once recruited, Tregs inhibit effector T cell responses through the secretion of IL-10 and TGF-β, metabolic competition for IL-2, and the expression of inhibitory immune checkpoints (Flavell et al., 2010).

Despite this immunosuppressive landscape, cytotoxic CD8^+^ T and NK cells remain major effectors of antitumor immunity, while dendritic cells (DCs) coordinate innate and adaptive responses through antigen uptake, processing, and presentation. However, sustained exposure to inhibitory cytokines and tumor-derived soluble mediators progressively compromises DC maturation and antigen-presenting capacity, while impairing the cytotoxic function and fitness of CD8^+^ T and NK cells, ultimately favoring immune exhaustion and tumor immune escape (Spranger et al., 2015).

This impairment is further reinforced by soluble and matrix-associated factors released within the TME. In particular, matrix metalloproteinases (MMPs) and prostaglandin E2 (PGE2) reduce the expression of activating receptors on NK cells, including NKG2D and DNAM-1, thereby limiting their cytotoxic activity (Balsamo et al., 2009; López-Cobo et al., 2018). These findings illustrate how signals originating from different TME compartments converge on effector immune cells to progressively reduce their ability to recognize and eliminate melanoma cells.

Importantly, this immunoregulatory network is not composed exclusively of immunosuppressive factors. Several soluble mediators display marked functional duality, with their biological effects depending on the cellular and spatial context in which they operate. TGF-β provides a representative example: while contributing to immune homeostasis and tissue repair under physiological conditions, its persistent activation in melanoma is co-opted to suppress cytotoxic immunity and promote immune escape (Sheikh et al., 2025). Thus, rather than acting as intrinsically pro- or anti-tumoral signals, soluble mediators acquire distinct functions according to the microenvironmental context in which they are produced and received.

Beyond canonical immunosuppressive mechanisms, stromal and immune compartments actively regulate tumor immunogenicity and the visibility of melanoma cells to the immune system (Chen and Mellman, 2017; Binnewies et al., 2018). A central mechanism involves the NKG2D axis. NKG2D is an activating receptor expressed on NK cells and cytotoxic CD8^+^ T lymphocytes that recognizes stress-induced ligands, including MICA/B and UL16-binding proteins (ULBPs), expressed on tumor cells (Lanier, 2015). Within the melanoma TME, CAFs, TAMs, and matrix-remodeling enzymes can contribute to the downregulation or shedding of these ligands, thereby reducing immune recognition and cytotoxic responsiveness (Raulet et al., 2013). In particular, ADAM10/17-mediated proteolytic cleavage of MICA/B generates soluble ligands that decrease their cell-surface availability while promoting NKG2D desensitization on effector cells (Chitadze et al., 2013; Di Donato et al., 2025). These mechanisms collectively impair NK- and T-cell-mediated cytotoxicity and facilitate immune escape.

The NKG2D axis illustrates how the TME can modulate tumor visibility; however, immune regulation also depends on signaling pathways whose effects vary according to their intensity, duration, and spatial context. The stimulator of interferon genes (STING) pathway provides a representative example. Acute STING activation promotes type I interferon production, dendritic-cell maturation, antigen presentation, and effective T-cell priming. Conversely, persistent or spatially restricted STING activation may sustain chronic inflammation, immune exhaustion, and resistance to immune checkpoint blockade, particularly within suppressive myeloid-rich niches (Woo et al., 2014; Corrales et al., 2015; Sivick et al., 2018; Li et al., 2019). Thus, the same signaling pathway may support antitumor immunity under acute conditions but become maladaptive when persistently activated within a suppressive microenvironment.

Importantly, these immunoregulatory programs are not uniformly distributed throughout melanoma lesions. Spatial transcriptomic analyses indicate that immune suppression can be organized into localized niches characterized by suppressive myeloid populations, dysfunctional lymphocytes, and inhibitory cytokine gradients (Joyce and Fearon, 2015; Jerby-Arnon et al., 2018). These spatially defined microenvironments may therefore determine where immune recognition is maintained or lost within the tumor. Moreover, immunoregulatory circuits do not act independently of other TME compartments but can reshape vascular, stromal, and metabolic programs, supporting the view that immune escape is an emergent property of the melanoma ecosystem rather than the consequence of isolated immune defects.

## 4 The vascular axis: Blood vessels, lymphatics, and neural signaling

### 4.1 Angiogenesis and vascular dysfunction

Melanoma progression and metastatic dissemination depend also on dynamic vascular adaptation. Angiogenesis emerges from coordinated signaling exchanges between tumor, stromal, and endothelial compartments and is initiated by hypoxic stress (Leone et al., 2024).

Physiological angiogenic programs are essential for tissue repair and vascular homeostasis. However, within the melanoma TME, persistent hypoxia and chronic vascular endothelial growth factor (VEGF) signaling convert this tightly regulated process into pathological angiogenesis characterized by vascular dysfunction and immune exclusion. Although the VEGF/vascular endothelial growth factor receptor (VEGFR) axis remains the principal angiogenic driver, its activity is reinforced by a redundant network of soluble mediators, including fibroblast growth factor 2 (FGF-2), platelet-derived growth factor (PDGF), IL-8, and angiopoietins, which collectively sustain vascular remodeling and adaptation in melanoma (Wu et al., 2022; Cazzato et al., 2024).

TECs acquire aberrant phenotypic features that generate a dysfunctional and hyperpermeable vasculature characterized by leakiness, irregular perfusion, elevated interstitial pressure, and persistent hypoxia (Viallard and Larrivée, 2017). Such dysfunction perpetuates hypoxic niches that favour melanoma invasion while limiting effective immune cell infiltration (Zheng et al., 2025). Beyond classical sprouting angiogenesis, melanoma also exploits vasculogenic mimicry, in which aggressive tumor cells acquire endothelial-like features to establish alternative perfusion networks. Driven by hypoxia and endothelial-associated molecules, such as vascular endothelial cadherin (VE-cadherin) and ephrin type A receptor 2 (EphA2), this process bypasses canonical endothelial-dependent vascularization and contributes to resistance against anti-angiogenic therapies (Serratì et al., 2025).

Mast cells further reinforce this redundant angiogenic network by releasing canonical pro-angiogenic factors, including VEGF, FGF-2, and IL-6, as well as proteases such as tryptase, chymase, and matrix metalloproteinases that contribute to vascular and ECM remodeling (Cerro et al., 2022).

Importantly, endothelial cells also act as gatekeepers of immune accessibility. Autophagy-competent TECs and angiopoietin 2 (ANGPT2)/tyrosine kinase with immunoglobulin like and EGF- like domains 2 (TIE2) signaling actively limit T-cell infiltration, whereas disruption of endothelial autophagy enhances immune accessibility (Rigamonti et al., 2014; Leong and Kim, 2020; Verhoeven et al., 2023). Spatial transcriptomic mapping further demonstrates that vascular dysfunction generates compartmentalized hypoxic regions that locally reinforce angiogenic signaling, dedifferentiation programs, and immune exclusion. These observations highlight that vascular dysfunction is not an isolated angiogenic phenomenon, but a spatially organized process that links hypoxia, immune exclusion, and melanoma cell-state plasticity.

Importantly, angiogenic mediators illustrate the context-dependent nature of soluble signaling within the melanoma ecosystem. Molecules such as VEGF are indispensable for physiological vascular homeostasis and tissue repair but become potent drivers of vascular dysfunction, immune exclusion, and therapeutic resistance when persistently activated within the tumor microenvironment (Wu et al., 2022). Thus, their biological impact depends not simply on the level of expression, but on the spatial and temporal context in which these signals are integrated with other TME-derived cues. Collectively, these observations indicate that the vascular compartment functions not merely as a conduit for oxygen and nutrient delivery, but as a dynamic signaling interface that continuously communicates with immune, stromal, and tumor compartments. Through reciprocal exchange of soluble mediators, vascular remodeling integrates hypoxia, immune exclusion, ECM remodeling, and melanoma cell-state plasticity, thereby contributing to progression and therapeutic resistance.

### 4.2 Lymphatic remodeling and immune trafficking

The cutaneous lymphatic vasculature constitutes one of the principal structural landmarks of the skin, where it maintains tissue-fluid homeostasis, antigen drainage, and immune surveillance under physiological conditions (Chen et al., 2026). Owing to its strategic anatomical position at the interface between peripheral tissues and draining lymph nodes, the lymphatic network continuously coordinates local and systemic immune communication. During melanoma progression, however, tumor-derived soluble mediators transform the lymphatic vasculature into a dynamic signaling interface that supports melanoma plasticity, immune escape, and metastatic dissemination (Suman and Markovic, 2023; Takeda et al., 2023).

Tumor-associated lymphangiogenesis is predominantly orchestrated by the VEGF-C/VEGF-D–VEGFR3 axis. Beyond regulating lymphatic drainage, VEGF-C signaling shapes antigen transport, chemokine gradients, and immune-cell trafficking within the TME. VEGF-C-driven lymphangiogenesis promotes cross-presentation of tumor antigens by lymphatic endothelial cells (LECs), resulting in dysfunctional activation and deletion of antigen-specific CD8^+^ T cells (Lund et al., 2012). Conversely, under specific therapeutic conditions, VEGF-C-induced lymphangiogenesis enhances CCL21-dependent recruitment of naïve T cells, thereby increasing intratumoral T-cell infiltration and potentiating immune checkpoint blockade (Fankhauser et al., 2017). Collectively, there is a dual and context-dependent nature of lymphatic remodeling. Thus, lymphangiogenesis exemplifies the behavior of soluble mediator networks proposed throughout this review, simultaneously promoting immune activation or immune tolerance according to the evolving microenvironmental context.

Consistently, in addition to coordinate antigen transport and immune-cell trafficking between the primary tumor and draining lymph nodes (Takeda et al., 2023), LECs can modulate immune-cell function through metabolic signals. Lymphatic-derived 25-hydroxycholesterol (25-HC), for example, restrains PPAR-γ-dependent immunosuppressive polarization of macrophages and monocytes, thereby enhancing effector T-cell responses and responsiveness to immunotherapy (Sun and Dong, 2026). Thus, lymphatic remodeling extends beyond vessel expansion and immune trafficking to integrate metabolic and immunoregulatory signals within the melanoma ecosystem.

Melanoma-derived EVs reinforce this reciprocal crosstalk. EV-associated nerve growth factor receptor (NGFR) stimulates lymphangiogenesis through ERK/NF-κB activation and ICAM-1 upregulation, thereby enhancing melanoma cell adhesion to the lymphatic endothelium (García-Silva et al., 2021). Simultaneously, EV uptake by LECs within tumor-draining lymph nodes induces their transcriptional reprogramming while delivering tumor antigens for major histocompatibility complex class I (MHC-I) cross-presentation, promoting early immune remodeling and contributing to the establishment of a pre-metastatic niche (Leary et al., 2022). Within this adaptive lymphatic niche, CCR7-expressing melanoma cells exploit LEC-derived CCL21 gradients to migrate toward lymphatic vessels and draining lymph nodes (Shields et al., 2007; Takeda et al., 2023). Accordingly, the sentinel lymph node is increasingly recognized not merely as the first anatomical site of metastatic dissemination but as an early immunologically remodeled ecosystem in which soluble mediators, EVs, and lymphatic reprogramming cooperate to shape immune responses before overt metastatic colonization (Gillot et al., 2021).

In such a manner, we can identify the lymphatic compartment not as an inert bystander during melanoma progression but as an active node within a dynamic signaling network that continuously reshapes the tumor ecosystem.

### 4.3 Peripheral innervation and neuroimmune crosstalk

Together with blood and lymphatic vessels, peripheral nerves constitute one of the three major structural networks of the skin. Peripheral nerves continuously transmit biochemical information thereby actively shaping tissue homeostasis and tumor adaptation (Ständer and Schmelz, 2024). The relationship between melanoma and the peripheral nervous system is further supported by their shared neural crest origin, which may partly explain the remarkable responsiveness of melanoma cells to neurotrophic cues (Adameyko et al., 2009). Nerve fibers relate to tumor, stromal, vascular, and immune cells through the release of neurotransmitters, neuropeptides, neurotrophic factors, cytokines, and lipid mediators (Sun et al., 2026). In addition, melanoma exploits these physiological neuroimmune communication pathways to promote phenotypic plasticity, immune escape, and metastatic dissemination (Benboubker et al., 2022).

Melanoma actively remodels its neural microenvironment by inducing axonogenesis and increasing tumor innervation through the secretion of neurotrophic factors, particularly NGF and brain-derived neurotrophic factor (BDNF) (Giampietri et al., 2025). In turn, neural derived soluble mediators regulate angiogenesis, inflammatory responses, and immune cell activity, generating reciprocal signaling circuits that continuously reshape the tumor (Shalabi et al., 2024). Importantly, neural remodeling should not be considered a secondary consequence of tumor expansion but rather an active component of melanoma progression (Ständer and Schmelz, 2024).

Interestingly, the biological effects of innervation are highly context-dependent thus closely recapitulating the dual behavior of soluble mediators described throughout this review. Sympathetic signaling generally promotes tumor progression through catecholamine-mediated enhancement of angiogenesis, chronic inflammation, and immunosuppressive programs. In contrast, sensory neurons appear to exert protective functions in melanoma. Experimental sensory denervation accelerates melanoma growth and intratumoral angiogenesis, whereas activation of nociceptive neurons restrains tumor progression and enhances anti-tumor immunity. Consistently, increased expression of sensory neuron-associated genes correlates with improved patient survival, suggesting that distinct neural subsets exert divergent effects on melanoma evolution (Costa et al., 2021). This functional duality closely mirrors the context-dependent behavior of several soluble mediators discussed throughout this review.

Among the neural-associated stromal cells, Schwann cells have recently emerged as central orchestrators of this neuroimmune crosstalk (Rinwa et al., 2021). In response to melanoma-derived signals, Schwann cells undergo a repair-like reprogramming reminiscent of peripheral nerve injury, acquiring migratory, trophic, and immunomodulatory properties. However, unlike physiological nerve repair, where this transient phenotype promotes tissue regeneration and resolves inflammation, tumor-associated Schwann cells remain chronically activated, sustaining an immunosuppressive microenvironment (Giampietri et al., 2025). This persistent repair-like phenotype illustrates how melanoma co-opts physiological tissue regeneration programs to establish an immunosuppressive niche that favors tumor persistence. Reprogrammed Schwann cells release a broad repertoire of soluble mediators, including CCL2, CXCL5, IL-33, PGE2, glial cell line-derived neurotrophic factor (GDNF), and additional neurotrophic factors that recruit suppressive myeloid populations, remodel the extracellular matrix, facilitate tumor cell migration, and promote perineural dissemination (Yang et al., 2026).

Neuroimmune communication further contributes to immune evasion (Guo et al., 2026). Melanoma-induced activation of nociceptor neurons stimulates the release of calcitonin gene-related peptide (CGRP), which suppresses CD8^+^ T-cell cytotoxicity and favors the establishment of an immunosuppressive microenvironment (Balood et al., 2022). Likewise, NGF promotes immune cell exclusion and impairs the generation of durable anti-tumor T-cell memory, whereas pharmacological inhibition of NGF restores sensitivity to immune checkpoint blockade and enhances longterm immune surveillance (Yin et al., 2024). These findings identify neurotrophic signaling not only as a regulator of melanoma progression but also as a promising therapeutic target.

## 5 The mechanotransducive and ECM axis: Structural remodeling

While transient ECM remodeling is an essential, self-limiting feature of physiological tissue repair, melanoma continuously subverts this program. The persistent ECM activation transforms a controlled tissue-healing response into a chronic, deregulated process that actively promotes tumor invasion, immune exclusion, and therapeutic resistance. (Lu et al., 2012; Botti et al., 2013; Popovic and Tartare-Deckert, 2022).

ECM remodeling is sustained by an imbalance between MMPs, particularly MMP-2 and MMP-9 and their endogenous tissue inhibitors (TIMPs) resulting in continuous matrix turnover (Kessenbrock et al., 2010; Pittayapruek et al., 2016). This process is fueled by tumor- and stroma-derived soluble factors, predominantly TGF-β, which reshape tissue organization and increase matrix stiffness.

While keratinocytes primarily influence melanocyte behavior during the early stages of melanoma genesis through paracrine signaling, CAFs emerge as dominant regulators of ECM remodeling and stromal reprogramming (Mazurkiewicz et al., 2021). Crucially, as revealed by single-cell resolution studies, specific subpopulations, such as myCAFs, spatially orchestrate matrix stiffening to create physical barriers against immune infiltration. CAF subsets integrate inflammatory, mechanical, and metabolic signals, thereby coordinating the reciprocal interactions between stromal remodeling and melanoma cell-state transitions. Through the localized secretion of TGF-β, IL-6, and CXCL12, CAFs establish reciprocal communication networks that promote invasive and dedifferentiated melanoma states (Mao et al., 2021; Zhou et al., 2024). Importantly, TGF-β exemplifies the context-dependent behavior of soluble mediators. Under physiological conditions it contributes to tissue repair and matrix homeostasis. Persistent activation, instead, drives pathological fibrosis, immune suppression, and invasive cell-state transitions. Progressive matrix stiffening not only increases the physical resistance of the tumor tissue but also activates mechanosensitive signaling pathways that reinforce invasive phenotypes, limit immune cell infiltration, and reduce drug accessibility. Thus, mechanical remodeling becomes tightly integrated with immune and vascular dysfunction rather than representing an isolated physical barrier. (Pickup et al., 2014).

Mechanically induced activation of YAP/TAZ and related mechanosensitive transcriptional programs further reinforce melanoma dedifferentiation, survival, and therapeutic resistance (Gargalionis et al., 2023).

These observations redefine the tumor matrix as a responsive interface in which biomechanical and soluble cues are continuously integrated. Through feedback with the secretome, force-activated pathways influence immune-cell positioning, vascular organization, and melanoma cell-state transitions, collectively reinforcing the malignant ecosystem under therapeutic stress.

## 6 The metabolic and redox axis: Adapting to microenvironmental stress

Metabolic adaptation extends far beyond bioenergetic requirements and has emerged as a major signaling mechanism coordinating melanoma plasticity. Within the TME, metabolites function as *bona fide* soluble mediators (Pavlova and Thompson, 2016). CAFs and adipocytes provide essential metabolic support. CAFs engage in direct organelle transfer, including mitochondrial exchange, while regulating enzymatic activity to facilitate the exchange of metabolites such as lactate, amino acids, and fatty acids (Avagliano et al., 2020). Adipocytes also contribute to melanoma progression by providing a metabolically active reservoir within the subcutaneous microenvironment. As melanoma cells invade beyond the dermo-epidermal barrier and reach adipose tissue, reciprocal interactions with adipocytes induce changes in adipocyte morphology and metabolic function, increasing lipid availability and supporting tumor metabolic adaptation and invasive behavior (Zhang et al., 2018). Importantly, these metabolic interactions extend beyond tumor cell survival and invasion to influence immune function. Alterations in nutrient availability within the TME can directly impair antitumor immune responses; for instance, CAF-mediated depletion of L-arginine compromises CD8^+^ T-cell cytotoxicity and thereby contributes to immune evasion (Geiger et al., 2016; Érsek et al., 2020). Thus, stromal metabolic remodeling links nutrient exchange with immune dysfunction, illustrating how metabolic signals generated within the TME can simultaneously support melanoma adaptation and suppress antitumor immunity. The major TME-driven adaptive programs that emerge from metabolic and redox remodeling and contribute to melanoma therapeutic resistance are summarized in Table 3.

**TABLE 3:** TME-driven adaptative programs contributing to melanoma therapeutic resistance.

TME program	Key cellular components	Major soluble mediators/Pathway axes	Mechanistic contribution to resistance	Therapy context	References
Paracrine rescue and signaling redundancy	Cancer-associated fibroblasts (CAFs), melanoma cells, stromal cells	HGF-MET, IL-6/STAT3, FGF, TGF-β	Activation of compensatory signaling pathways (MAPK, PI3K/AKT, STAT3, NF-kB), enabling tumor survival under therapeutic pressure	Mainly BRAF/MEK inhibitors; contributes to adaptive resistance to ICIs	(Kakadia et al., 2018; Falcone et al., 2020)
Immunosuppressive microenvironment and immune exclusion	Tumor -associated macrophages (TAMs), myeloid-derived suppressor cells (MDSCs), regulatory T cells (Tregs), dysfunctional dendritic cells	TGF-β, IL-10, VEGF, arginase-1	Suppression of effector T-cell activity; impaired antigen presentation and recruitment of effector lymphocytes, establishment of immune-excluded tumor phenotype	Primary and acquired resistance to ICIs	(Smith et al., 2014; Spranger et al., 2015; Simiczyjew et al., 2020)
Angiogenesis and vascular remodeling	Tumor endothelial cells (TECs), pericytes, stromal cells	VEGFR, ANGPT2/TIE2, IL-8	Reactivation of pro-angiogenic signaling maintains dysfunctional vasculature, hypoxia and limited drugs delivery	Immunotherapy and target therapy	(Wu et al., 2022; Cazzato et al., 2024)
Vasculogenic mimicry	Melanoma Cells	Hypoxia, ECM signaling	Formation of vascular like channels independent of endothelial cells, enabling escape from anti-angiogenic therapies	Targeted therapy and anti-angiogenic therapy	(Serratì et al., 2025)
ECM remodeling and stromal stiffening	Cancer-associated fibroblasts (CAFs), ECM components (collagens, fibronectin)	TGF-β, MMP-2/9, integrin/FAK/SRC	Increase ECM deposition and matrix stiffness reduce immune infiltration, limit drug penetration and activate pro-survival mechanotransduction pathways	Immunotherapy and target therapy	(Almeida et al., 2019; Jurj et al., 2022)
Metabolic and redox adaptation	Tumor cells, fibroblasts, endothelial cells	HIF-1α lactate, ROS signaling	Hypoxia and oxidative stress reshape tumor metabolism and modulate MAPK and PI3K signaling, supporting resistance	Immunotherapy and targeted therapy	(Pavlova and Thompson, 2016; Lee et al., 2020)
Therapy-induced TME adaptive rewiring	Integrated tumor-stroma-immune network	PD-L1, pro-angiogenic cytokines, chemokines	Dynamic microenvironmental reconfiguration under therapeutic pressure promoting immune exclusion, vascular instability, stromal reinforcement and metabolic adaptation	Immunotherapy and BRAF/MEK inhibitors	(Proietti et al., 2020; Turner et al., 2025)

Tumor-derived metabolites include lactate, lipids and amino acids derivatives (Sun et al., 2022). Lactate, generated by enhanced aerobic glycolysis, has emerged as one of the principal metabolic mediators coordinating tumor-induced immunosuppression (Zheng et al., 2024; Gu et al., 2025). By interfering with the glycolytic flux required for T-cell activation and effector function, lactate suppresses T-cell proliferation, reduces IL-2 and IFN-γ production, and impairs cytotoxic activity (Fischer et al., 2007). Simultaneously, by the extracellular acidification, it promotes T-cell exhaustion with a reduced perforin and granzyme expression and a defective lytic granule exocytosis (Zheng et al., 2024). Beyond its effects on cytotoxic T lymphocytes, lactate broadly reshapes also innate immunity. It impairs DCs maturation, promotes the polarization of TAMs toward an immunosuppressive phenotype, suppresses the inflammatory functions of M1-like macrophages, and reduces NK-cell cytotoxicity, IFN-γ production, infiltration, and survival, thereby reinforcing a tolerogenic microenvironment (Dempsey, 2014; Jedlička et al., 2022; Gu et al., 2025). Importantly, lactate also exemplifies the context-dependent nature of soluble mediators. While it serves as an essential physiological metabolite during normal tissue homeostasis, its persistent accumulation within the melanoma TME coordinates immune suppression, angiogenesis, and stress adaptation. In addition, lactate regulates gene expression through histone lactylation, increasing ST2 receptor expression, enhancing IL-33 responsiveness, and promoting pro-angiogenic programs, thereby extending its influence beyond metabolism into epigenetic and microenvironmental remodeling (Dempsey, 2014; Pascual et al., 2017; Wu and Näär, 2019).

In addition to glycolytic rewiring, melanoma cells display remarkable lipid metabolic flexibility by increasing fatty acid uptake, *de novo* lipogenesis, and fatty acid oxidation (FAO), thereby adapting to metabolic stress while sustaining energetic homeostasis, invasive plasticity, and therapeutic resistance (Pascual et al., 2017; Wu and Näär, 2019; Du et al., 2026). Lipid accumulation and nutrient competition impair immune cell metabolism, promoting T-cell dysfunction and the establishment of an immunosuppressive, tolerogenic niche (Pellerin et al., 2020). Furthermore, FAO-dependent metabolic programs intersect with autophagy and stress-adaptive pathways, enhancing melanoma cell survival, migration, and metastatic dissemination under hostile microenvironmental conditions. Accordingly, disruption of autophagy impairs tumor cell invasiveness and reduces metastatic potential (Shin et al., 2023).

In parallel, amino acid metabolism further contributes to immune dysfunction through the depletion of essential nutrients, such as arginine and tryptophan, and the accumulation of immunoregulatory metabolites, including kynurenine, which suppress effector T-cell function and reinforce tolerogenic signaling within the tumor microenvironment (Capece et al., 2010; Mezrich et al., 2010; Chevolet et al., 2015; Chen et al., 2024).

### 6.1 ER stress and the UPR: Integrating stress adaptation, immune dysfunction, and therapeutic resistance

Beyond metabolic rewiring, oxidative and endoplasmic reticulum (ER) stress have emerged as central mechanisms through which melanoma coordinates adaptive responses across the entire TME. For example, senescent fibroblasts lose the expression of superoxide dismutase-3 (SOD3) and release factors associated with the senescence-associated secretory phenotype (SASP), altering reactive oxygen species (ROS) susceptibility in melanoma cells and sustaining stress-associated signaling pathways involved in tumor survival and progression (Oliveira et al., 2016; Pizzimenti et al., 2021).

Within the TME, oxidative stress and oxygen deprivation cooperate to stabilize hypoxia-inducible factors (HIFs), particularly HIF-1α, promoting interconnected adaptive programs that drive angiogenesis, ECM remodeling, metabolic rewiring, and therapeutic resistance (Ogier-Denis and Codogno, 2003; Bedogni and Powell, 2009; Bellot et al., 2009; Semenza, 2012; Gilkes et al., 2014; Daskalaki et al., 2018; Hu et al., 2022; Ni et al., 2025). In parallel, oxidative and ER stress establish a self-reinforcing adaptive circuit (Zhou et al., 2021) in which ROS accumulation (Ong and Logue, 2023) disrupts proteostasis and activates the unfolded protein response (UPR) (Sykes et al., 2016). Thus, hypoxia, nutrient deprivation, acidosis, and metabolic imbalance converge on stress-adaptive program that promotes melanoma survival while simultaneously reshaping the surrounding microenvironment (Hwang et al., 2025).

ER stress can extend beyond the stressed cell through transmissible ER stress (TERS). In such a way, cytokines, EVs, and damage-associated molecular patterns released by stressed melanoma cells can propagate ER-stress-associated signals to neighboring stromal and immune cells (Mahadevan et al., 2011; Jiang et al., 2020; Kalluri and LeBleu, 2020, Zarrella et al., 2025). ER stress activates the three canonical UPR branches (IRE1α/XBP1, PERK, and ATF6), collectively reshaping proteostasis, cytokine production, oxidative stress tolerance, and antigen presentation (Amodio et al., 2018; Bartoszewski et al., 2020; Hwang et al., 2025). As consequences there are also the tolerogenic myeloid states, the T-cell and NK-cell dysfunction and in general the occurrence of an immunosuppressive microenvironment (Wang and Kaufman, 2014; Cubillos-Ruiz et al., 2015; Kim et al., 2018; Lebeaupin et al., 2020; Chen and Cubillos-Ruiz, 2021; Hwang et al., 2025). Thus, UPR signaling exemplifies the dual nature of stress-associated mediators. While transient activation preserves tissue homeostasis by restoring proteostasis under physiological conditions, persistent activation within the melanoma TME is co-opted to sustain immune suppression, metabolic adaptation, and therapeutic resistance.

More in detail, in DCs, activation of the IRE1α/XBP1 pathway leads to lipid accumulation, impairing antigen presentation, and consequently reducing T cell activation (Cubillos-Ruiz et al., 2015). In MDSCs and macrophages, ER stress suppresses CD8^+^ and CD4^+^ T cells as well as NK cells, promoting an immunosuppressive TME (Cubillos-Ruiz et al., 2015; Kim et al., 2018; Hwang et al., 2025). In T lymphocytes, ER stress induces cellular exhaustion (Ma et al., 2019; Tang et al., 2022; He et al., 2024), while in NK cells, ER stress can impair cytotoxic function and tumor-cell recognition (Raulet et al., 2013; Chen and Cubillos-Ruiz, 2021). Emerging evidence suggests that stress-associated signaling pathways may also enhance the expression of NKG2D ligands, including ULBP1, thereby increasing melanoma susceptibility to NK-cell-mediated cytotoxicity and highlighting the dynamic and reversible nature of tumor immunogenicity (Sorrentino et al., 2026). These apparently divergent observations further highlight the context-dependent nature of stress signaling, suggesting that ER stress can either impair or enhance tumor immunogenicity according to the intensity, duration, and cellular context of UPR activation.

## 7 Adaptive plasticity and therapeutic resistance: Stress-driven reprogramming of the melanoma ecosystem

Despite the remarkable advances achieved with targeted therapies and ICIs, durable clinical responses remain limited by adaptive therapeutic resistance (Somasundaram et al., 2016; Florent et al., 2023; Serratì et al., 2025; Sorrentino et al., 2026). Resistance reflects the dynamic interplay between melanoma cells and the surrounding TME (Avagliano et al., 2020; Dilshat et al., 2021; Najem et al., 2022; Turner et al., 2025).

Melanoma plasticity is increasingly sustained by reversible transcriptional, epigenetic, metabolic, and stress-adaptive reprogramming (Dilshat et al., 2021; Najem et al., 2022). This adaptive flexibility generates heterogeneous tumor cell states capable of rapidly responding to therapeutic pressure and microenvironmental cues (Avagliano et al., 2020; Najem et al., 2022; Sabău et al., 2025; Turner et al., 2025). Consequently, therapeutic resistance should no longer be viewed simply as the outcome of clonal selection but rather as a dynamic process reinforced by reciprocal tumor–microenvironment interactions.

Tumor-intrinsic molecular alterations converge with microenvironment-derived signals onto key pathways, including MAPK, PI3K/AKT, MITF, NF-κB, and STAT3, thereby maintaining pro-survival programs despite pharmacological inhibition (Wu and Dai, 2017). Integration of these apparently distinct adaptive mechanisms is mediated by soluble mediator networks, which coordinate reciprocal communication among the different compartments. By synchronizing multiple biological programs under therapeutic stress, these networks drive phenotypic remodeling of both melanoma cells and the surrounding microenvironment, emerging as central regulators of tumor persistence rather than simple downstream effectors of therapeutic resistance.

From an immunoregulatory perspective, CAFs, TAMs, MDSCs, and dysfunctional lymphocyte populations establish interconnected soluble mediator networks that impair antigen presentation, suppress cytotoxic T-cell and NK-cell activity, and promote immune exclusion. CAF-derived TGFβ represents a central integrative hub linking immune suppression with stromal remodeling by promoting MITF-low/AXL-high dedifferentiated phenotypes while simultaneously driving extracellular matrix remodeling and matrix stiffening (Falcone et al., 2020; Lee et al., 2020). In addition, prostaglandin E2, matrix-remodeling enzymes, and MMP-9-mediated PD-L1 regulation further compromise immune checkpoint blockade by reducing NK-cell activation and reinforcing adaptive immune resistance (Balsamo et al., 2009; López-Cobo et al., 2018). Spatially, these suppressive programs generate immune-excluded niches enriched in dysfunctional myeloid populations and exhausted lymphocytes, providing a permissive environment for therapeutic escape.

The vascular axis further contributes to therapeutic failure by limiting both drug delivery and immune-cell infiltration. Dysfunctional angiogenesis, persistent hypoxia, and vasculogenic mimicry cooperate to maintain poorly perfused tumor regions in which adaptive hypoxia-associated signaling programs are continuously reinforced (Cerro et al., 2022; Serratì et al., 2025). These processes establish specialized microenvironmental niches that protect melanoma cells from therapeutic pressure while promoting immune exclusion and metabolic adaptation.

From a mechanotransductive perspective, the tumor stroma actively regulates melanoma adaptation through reciprocal biochemical and biomechanical signaling. CAF-mediated ECM remodeling, increased matrix stiffness, and activation of integrin-β1/FAK/SRC and YAP/TAZ signaling sustain adaptive melanoma states while limiting drug penetration. In parallel, HGF-MET signaling maintains MAPK and PI3K/AKT activation despite BRAF inhibition, illustrating how stromal-derived soluble mediators compensate for pharmacological blockade of tumor-intrinsic pathways (Almeida et al., 2019; Falcone et al., 2020).

The metabolic-redox axis provides an additional layer of adaptive plasticity. Nutrient depletion, oxidative stress, ROS accumulation, hypoxia, ER stress collectively promote stress tolerance, metabolic flexibility, and immune dysfunction (Cubillos-Ruiz and Glimcher, 2015; Almeida et al., 2019; Pizzimenti et al., 2021; Sabău et al., 2025).

Collectively, these pathways enhance melanoma survival while reshaping the immune landscape under therapeutic pressure. Thus, these biological programs generate spatially organized resistant niches, which provide the biological substrate for reversible melanoma cell-state transitions and the emergence of therapy-resistant populations.

This framework provides a biological rationale for developing combinatorial therapeutic strategies aimed at disrupting adaptive communication networks rather than individual tumor-intrinsic pathways. The principal tumor microenvironment-driven mechanisms contributing to therapeutic resistance are summarized in Table 3 and visually represented in Figure 2.

**FIGURE 2:**
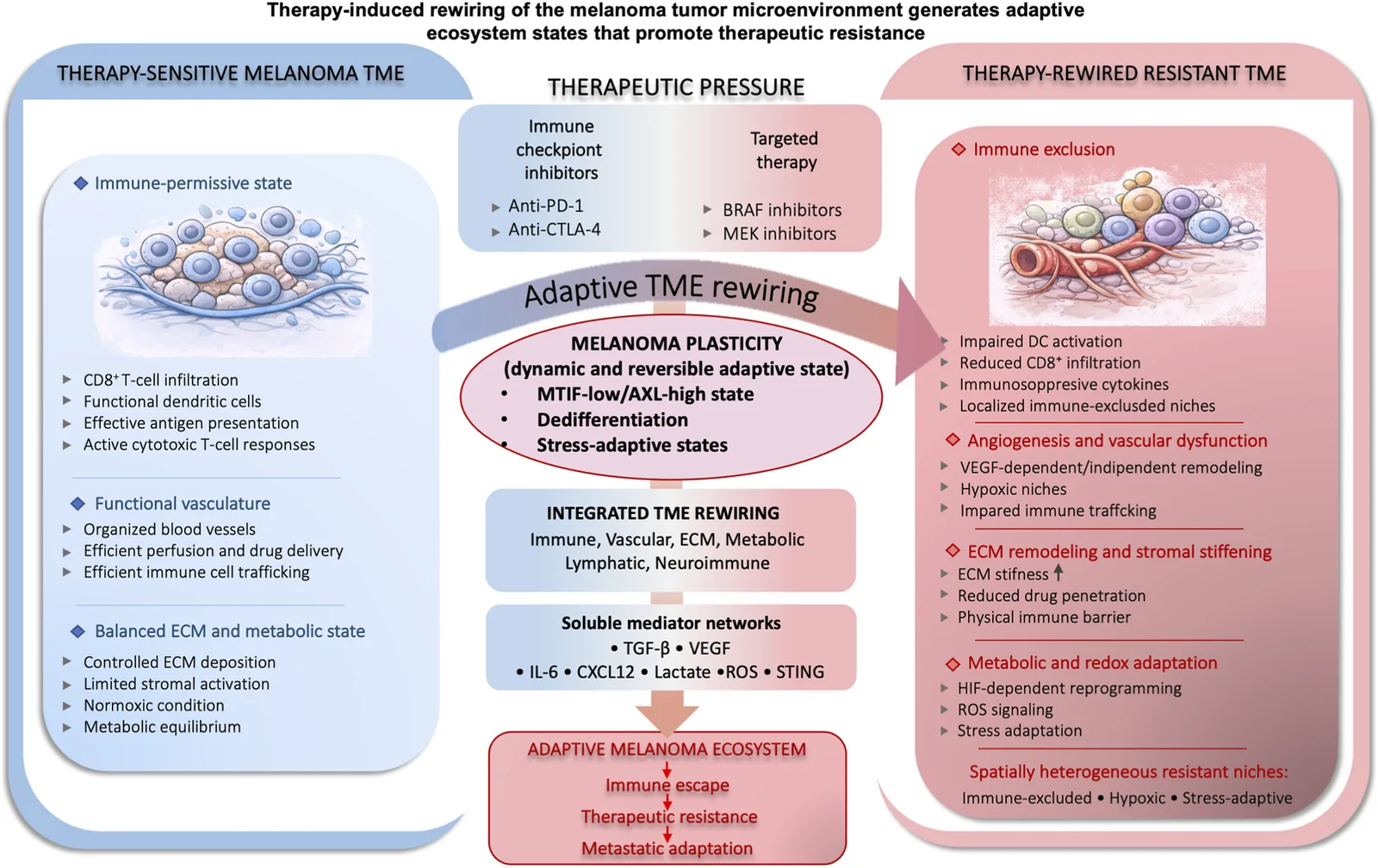
Therapy-induced rewiring of the melanoma tumor microenvironment drives adaptive resistance. This schematic illustrates the transition from a therapy-sensitive to a therapy-rewired resistant melanoma tumor microenvironment (TME). Therapeutic pressure induces reversible melanoma cell plasticity together with coordinated remodeling of immune, vascular, extracellular matrix (ECM), metabolic, lymphatic, and neuroimmune programs, integrated through interconnected soluble mediator networks. Continuous reciprocal interactions among melanoma cells, stromal cells, immune populations, endothelial and lymphatic endothelial cells, and ECM components progressively reshape the tumor ecosystem, generating spatially heterogeneous resistant niches characterized by immune exclusion, vascular dysfunction, hypoxia, ECM remodeling, and metabolic adaptation. Collectively, these interconnected programs drive immune escape, therapeutic resistance, and metastatic adaptation, highlighting that these clinically relevant outcomes emerge as ecosystem-level properties of continuous tumor–microenvironment communication.

## 8 Organ-specific reconfiguration of the four biological axes during melanoma metastatic progression

During metastatic dissemination, melanoma cells leave the primary cutaneous niche and colonize different tissues. Metastatic melanoma continuously reconfigures the same adaptive signaling networks in response to tissue-specific soluble mediators and local microenvironmental constraints. Consequently, metastatic progression should be viewed as a dynamic process in which organ-specific cues selectively reinforce different adaptive programs while preserving the integrated signaling architecture described throughout this review (Massagué and Obenauf, 2016).

Among metastatic sites, the brain represents one of the most specialized ecosystems. The bloodbrain barrier, resident astrocytes and microglia, together with a lipid-rich metabolic environment, reinforce immunoregulatory and metabolic-redox programs, enabling immune evasion while supporting energetic flexibility (Klemm et al., 2020).

Compared with the primary cutaneous niche, the brain is characterized by a hyaluronan-rich, compliant ECM, which reshapes the biomechanical cues experienced by disseminated melanoma cells. Consequently, successful metastatic colonization relies less on adaptation to a rigid stromal environment and more on the ability of melanoma cells to rapidly reprogram metabolic and immunoregulatory circuits. To meet these tissue-specific demands, melanoma cells exploit the lipid-rich cerebral milieu while simultaneously releasing soluble mediators that reprogram resident astrocytes and microglia into a neuroinflammatory immunosuppressive niche. This reciprocal crosstalk limits cytotoxic T-cell infiltration, promotes immune evasion, and ultimately contributes to therapeutic resistance. These adaptive responses are coordinated by tissue-specific soluble mediator networks that are summarized in Table 4.

**TABLE 4:** Organ-specific features of the melanoma metastatic microenvironment.

Metastatic niche	Predominant biological axis	Dominant immune cells	Stromal/ECM features	Metabolic features	Key soluble mediators	References
Brain Metastatic Niche	Immunoregulatory and metabolic	Microglia, infiltrating MDSCs	Hyaluronan-rich soft ECM, blood-brain barrier, reactive astrocytes	Lipid-dependent adaptation, high OXPHOS reliance	TGF-β, Neurotrophins, STING-associated cytokines	(Boire et al., 2020; Klemm et al., 2020)
Lung Metastatic Niche	Vascular and metabolic-redox	Alveolar macrophages, neutrophils	Elastic ECM, dense capillary network, active fibroblast remodeling	High oxygen availability, profound oxidative stress	TGF-β, CXCL12	(Altorki and Lee, 2019; Fares et al., 2020)
Liver	Immunoregulatory	Kupffer cells, Regulatory T cells (Tregs)	Sinusoidal endothelium, hepatic stellate cells	Glutamine dependency, enhanced lipid metabolism	IL-10, TGF-β, HGF	(Brodt, 2016; Yu et al., 2021)
Bone	Mechanotransductive and Metabolic	Tregs, exhausted T cells	Mineralized rigid ECM, osteoclast/osteoblast imbalance	Hypoxic, highly glycolytic, lactate-rich	TGF-β, CXCL12	(Croucher et al., 2016; Fornetti et al., 2018)

Different adaptive solutions emerge within the lung, (Table 4), where melanoma cells encounter a highly oxygenated environment characterized by an extensive capillary network and an elastic ECM. These conditions increase oxidative stress, making metabolic-redox adaptation particularly important for metastatic survival (Fares et al., 2020). At the same time, reciprocal interactions with endothelial cells and pulmonary fibroblasts promote vascular remodeling and ECM reorganization (Pickup et al., 2014), while tumor-derived soluble mediators polarize resident alveolar macrophages toward immunosuppressive phenotypes. Together, these adaptive responses illustrate how vascular remodeling cooperates with metabolic redox and immunoregulatory programs to sustain metastatic persistence.

The liver provides a physiologically tolerogenic environment in which immunoregulatory soluble mediators (Table 4) amplify pre-existing mechanisms of immune tolerance. Here disseminated melanoma cells amplify pre-existing immunoregulatory mechanisms by interacting with Kupffer cells and sinusoidal endothelial cells, which produce soluble mediators including IL-10, TGF-β, and HGF. These signaling networks suppress CD8^+^ T-cell activity while promoting immune tolerance and metastatic outgrowth (Brodt, 2016; Sun et al., 2022). Concurrently, melanoma adapts to hepatic nutrient availability through increased lipid utilization and glutamine metabolism, illustrating how metabolic rewiring complements the dominant immunoregulatory landscape. Thus, the liver exemplifies how physiological tissue functions can be co-opted by melanoma to support metastatic fitness.

A distinct adaptive scenario is observed in bone, where soluble mediators involved in bone remodeling (Table 4) coordinate reciprocal communication between melanoma cells, osteoblasts, osteoclasts, and immune cells. The rigid mineralized ECM enhances mechanotransductive signaling, while reciprocal interactions with osteoblasts and osteoclasts, mediated by soluble factors including TGF-β and CXCL12, disrupt bone homeostasis and promote metastatic progression (Croucher et al., 2016; Fornetti et al., 2018). Progressive bone remodeling further generates hypoxic regions that favor glycolytic metabolism and lactate accumulation, linking biomechanical adaptation with metabolic rewiring and immune exclusion.

Rather than establishing organ-specific biological programs, metastatic dissemination continuously reshapes the same interconnected signaling framework in response to tissue-specific soluble mediator landscapes. The relative contribution of the different biological axes therefore changes according to the host tissue, generating distinct adaptive states while preserving the integrated ecosystem.

## 9 Conclusion

Advanced melanoma control remains still elusive for a proportion of patients (Marine et al., 2020; Fujisawa et al., 2021; Patel et al., 2021). The continuous bidirectional communication between tumor cells and their surrounding stromal, immune, vascular, lymphatic, neural, and metabolic compartments dictate disease evolution.

Thus, the principal conceptual contribution of this review is to propose that soluble mediator networks constitute the functional communication layer integrating these otherwise distinct biological programs. Rather than acting as isolated cytokines, chemokines, growth factors, metabolites, neurotrophic factors, stress-associated molecules, or ECM-derived signals, soluble mediators operate as highly interconnected signaling networks that synchronize immune regulation, angiogenesis, mechanotransduction, metabolic adaptation, lymphatic remodeling, and neuroimmune communication.

An equally important concept emerging from this review is the intrinsically context-dependent nature of soluble mediator signaling. Under physiological conditions, these molecules coordinate tissue homeostasis, immune surveillance, vascular integrity, lymphatic function, wound repair, and neuroimmune communication. During melanoma progression, however, persistent inflammatory stimuli, hypoxia, ECM remodeling, metabolic stress, and therapeutic pressure progressively rewire these same signaling pathways toward tumor-promoting functions. This dynamic functional duality represents the biological foundation of the “Bittersweet Symphony” proposed in the title of this review, emphasizing that melanoma evolution is driven not by individual molecules, but by the continuous adaptive reorganization of interconnected communication networks.

Importantly, this ecosystem perspective also redefines therapeutic resistance. It is not viewed exclusively as the consequence of genetically selected resistant clones but can be considered as an emergent property of a continuously adapting microenvironment in which reciprocal interactions among tumor cells and multiple stromal compartments generate spatially organized resistant niches. Recent advances in single-cell and spatial transcriptomics, spatial metabolomics, multiplex imaging, and other high-resolution technologies are now revealing these cellular and a-cellular compartments with unprecedented resolution, demonstrating that tissue-specific immune, vascular, stromal, neural, lymphatic, and metabolic programs cooperate to determine therapeutic response across both primary tumors and metastatic sites.

This integrated view now also offers a translational roadmap for future therapeutic development. Durable responses are unlikely to be achieved through inhibition of individual tumor intrinsic pathways alone. Instead, rational combinatorial therapeutic strategies capable of simultaneously disrupting multiple adaptive communication networks within the melanoma ecosystem are likely to represent the next-generation of therapeutic approaches. In this context, several soluble mediator pathways, including TGF-β, VEGF, the CXCL12/CXCR4 axis, STING signaling, immunometabolic pathways, and ER stress/UPR signaling, are currently under clinical investigation, largely in combination with ICIs or targeted therapies. Although these approaches are still evolving, they collectively support the concept that targeting interconnected soluble mediator networks may more effectively overcome adaptive resistance than inhibition of isolated signaling pathways. At this purpose, representative soluble mediator networks currently under clinical evaluation, together with their biological rationale and translational perspectives, are summarized in Table 5.

**TABLE 5:** Soluble mediator networks under clinical evaluation and their therapeutic rationale.

Soluble mediator	Biological axes involved	Therapeutic rationale	Combination strategy under investigation	Clinical perspective	Trial status	Study number and references
TGF-β	Tumor plasticity, CAF activation, ECM remodeling, immune suppression	TGF-β promotes immune evasion and reduces T-cell infiltration; inhibition may enhance immune checkpoint blockade	TGF-β receptor I inhibition (vactosertib) combined with PD-1 blockade (pembrolizumab) in acral and mucosal melanoma	Clinical evidence remains limited; however, this approach supports the rationale for co-targeting stromal and immune compartments involved in TGF-β-mediated immune exclusion	Phase II, completed; results not yet published	NCT05436990
VEGF	Angiogenesis; endothelial activation; vascular remodeling; metastasis	VEGF promotes angiogenesis and immune resistance; targeting this pathway may improve antitumor immunity	VEGFR inhibition with lenvatinib combined with temozolomide (DNA-alkylating agent) in advanced/metastatic melanoma	The observed antitumor activity supports the rationale for combining vascular and tumor cell-directed therapies, highlighting the contribution of the tumor microenvironment to treatment response	Phase I/Ib, completed; results published	NCT00121680 (Hong et al., 2015)
			VEGFR inhibition with lenvatinib combined with pembrolizumab (PD-1 blockade) as first-line treatment for advanced melanoma	Findings suggest that modulation of the vascular compartment did not translate into durable clinical benefit, highlighting the complexity of melanoma therapeutic resistance	Phase III, completed; results published	NCT03820986 (Arance et al., 2025)
			VEGFR inhibition with lenvatinib combined with dacarbazine (DNA-alkylating chemotherapy) as first-line treatment for stage IV melanoma	Results support the rationale for combining vascular targeting with cytotoxic therapy, reinforcing the role of tumor–stroma interactions in melanoma progression	Phase II, completed; results not yet published	NCT01133977
			VEGFR inhibition with lenvatinib combined with pembrolizumab (PD-1 blockade) after progression on prior PD-1/PD-L1 inhibitors in advanced melanoma	Clinical activity in PD-1/PD-L1-resistant melanoma supports further investigation of vascular targeting as part of combination strategies to address therapy-resistant disease	Phase II, completed; results published	NCT03776136 (Arance et al., 2023)
			Lenvatinib combined with pembrolizumab/quavonlimab (PD-1/CTLA-4 blockade) in advanced melanoma	This study investigates the potential of simultaneously targeting angiogenic and immune regulatory compartments; its clinical relevance remains to be established	Phase I/II, completed; results not yet published	NCT04305054
CXCL12/CXCR4	Immune exclusion; immunosuppression; tumor migration; stromal remodeling	CXCL12/CXCR4 signaling drives immune exclusion; inhibition may enhance immune cell infiltration	CXCR4 inhibition with mavorixafor (X4P-001) combined with pembrolizumab (PD-1 blockade) in advanced melanoma	Pharmacodynamic evidence of immune microenvironment remodeling supports targeting chemokine-mediated communication between stromal and immune compartments as a strategy to reduce immune exclusion	Phase Ib, completed; results published	NCT02823405 (Andtbacka et al., 2022)

Ultimately, the future of melanoma therapy will likely depend not on the identification of ever more individual molecular targets, but on the ability to disrupt the adaptive communication networks that continuously coordinate all the described programs across the tumor ecosystem. By placing soluble mediator networks at the center of melanoma biology, we propose a unified conceptual framework that links tumor plasticity, metastatic adaptation, and therapeutic resistance through a common biological language. We believe that this ecosystem-oriented perspective not only advances our understanding of melanoma progression but also provides a biologically grounded rationale for the design of next-generation combination therapies capable of simultaneously targeting multiple adaptive pathways, ultimately improving the durability of clinical responses in melanoma.

## Glossary

ADAM: A Disintegrin and Metalloproteinase
AKT: Rac-alpha serine/threonine-protein kinase
ANGPT2: Angiopoietin-2
ATF6: Activating transcription factor 6
AXL: AXL receptor tyrosine kinase
BDNF: Brain-derived neurotrophic factor
BCL-2: B-cell lymphoma 2
BiP: binding immunoglobulin protein
CAFs: Cancer-associated fibroblasts
CCL: Chemokine (C-C motif) ligand
CCR: C-C chemokine receptor
CD: Cluster of differentiation
CGRP: Calcitonin gene-related peptide
CTLA-4: Cytotoxic T-lymphocyte-associated protein 4
CXCL: Chemokine (C-X-C motif) ligand
DAMPs: Damage-associated molecular patterns
DCs: Dendritic cells
DNAM-1: DNAX accessory molecule-1
ECM: Extracellular matrix
EGF: Epidermal growth factor
EMT: Epithelial to mesenchymal transition
EphA2: Ephrin type A receptor 2
ER: Endoplasmic reticulum
EVs: Extracellular vesicles
FAK: Focal Adhesion Kinase
FAO: Fatty acid oxidation
FGF-2: Fibroblast growth factor 2
GDNF: Glial cell line-derived neurotrophic factor
HGF: Hepatocyte growth factor
HIFs: Hypoxia-inducible factors
iCAFs: Inflammatory cancer-associated fibroblasts
ICIs: Checkpoint inhibitors
IFN-γ: Interferon gamma
IL: Interleukin
IRE1α: Inositol-requiring enzyme 1 alpha
LAG-3: Lymphocyte activation gene-3
LECs: Lymphatic endothelial cells
MAPK: Mitogen-activated protein kinase
MCs: Mast cells
MDSCs: Myeloid-derived suppressor cells
MHC-I: Major histocompatibility complex class I
MICA/B: MHC class I polypeptide-related sequence A/B
MITF: Microphthalmia-associated transcription factor
MMPs: Matrix metalloproteinases
MSCs: Mesenchymal stromal cells
myCAFs: Myofibroblastic cancer-associated fibroblasts
NF-kB: Nuclear Factor kappa-light-chain-enhancer of activated B Cells
NGF: Nerve growth factor
NGFR: Nerve growth factor receptor
NK cells: Natural killer cells
NKG2D: Natural killer group 2 member D
NO: Nitric oxide
PCs: Pericytes
PD-1: Programmed cell death protein 1
PDGF: Platelet-derived growth factor
PD-L1: Programmed death-ligand 1
PERK: Protein kinase RNA-like endoplasmic reticulum kinase
PGE2: Prostaglandin E2
PI3K: Phosphoinositide 3-kinase
ROS: Reactive oxygen species
SASP: Senescence-associated secretory phenotype
scRNA-seq: Single-cell RNA sequencing
SOD3: Superoxide dismutase-3
SRC: proto-oncogene, non-receptor tyrosine kinase
ST2: Receptor serum stimulation-2
STAT3: Signal Transducer and Activator of Transcription 3
STING: Stimulator of interferon genes
TAMs: Tumor-associated macrophages
**TCF7** ^**+**^: Transcription factor 7 positive
TECs: Tumor-associated endothelial cells
TERS: Transmissible endoplasmic reticulum stress
TGF-β: Transforming growth factor beta
TIE2: Tyrosine kinase with immunoglobulin-like and EGF-like domains 2
TIM-3: T-cell immunoglobulin and mucin-domain containing-3
TIMPs: Tissue inhibitors of metalloproteinases
TME: Tumor microenvironment
TNF-α: Tumor Necrosis Factor alpha
**TOX** ^**+**^: Thymocyte selection-associated high mobility group box
Tregs: Regulatory T cells
ULBPs: UL16-binding proteins
UPR: Unfolded protein response
VE-cadherin: Vascular endothelial cadherin
VEGF: Vascular endothelial growth factor
VEGFR: Vascular endothelial growth factor receptor
XBP1: X-box binding protein 1
YAP/TAZ: Yes-associated protein/transcriptional coactivator with PDZ-binding motif
25-HC: Lymphatic-derived 25-hydroxycholesterol
